# Metal ion transport quantified by ICP-MS in intact cells

**DOI:** 10.1038/srep20551

**Published:** 2016-02-03

**Authors:** Julio A. Landero Figueroa, Cory A. Stiner, Tatiana L. Radzyukevich, Judith A. Heiny

**Affiliations:** 1 Department of Chemistry, University of Cincinnati, Cincinnati, OH USA; 2 University of Cincinnati/Agilent Technologies Metallomics Center of the Americas, Cincinnati, OH USA; 3 Department of Molecular and Cellular Physiology, University of Cincinnati, Cincinnati, OH USA

## Abstract

The use of ICP-MS to measure metal ion content in biological tissues offers a highly sensitive means to study metal-dependent physiological processes. Here we describe the application of ICP-MS to measure membrane transport of Rb and K ions by the Na,K-ATPase in mouse skeletal muscles and human red blood cells. The ICP-MS method provides greater precision and statistical power than possible with conventional tracer flux methods. The method is widely applicable to studies of other metal ion transporters and metal-dependent processes in a range of cell types and conditions.

Metal ion transport proteins are present in all eukaryotic cell membranes and perform vital cell functions. The transport rate of metal ion transporters, representing enzyme activity, is commonly determined from measurements of tracer flux. In a typical measurement, the cells of interest are incubated in a medium containing the transported ion and a trace amount of a radioactive isotope that is a congener for the transported ion. Transport rate is quantified as the amount of tracer taken up per unit time, after scaling by the molar ratio of tracer to the transported ion. The specific transport rate per molecule of enzyme is obtained by normalizing to the number of transporters in the sample, if known. Alternatively, flux in multicellular preparations is commonly normalized to cell number, tissue mass, or protein content. Efflux of ions by membrane transporters is assayed in a similar manner, after loading the cells with tracer. Radioactive tracer flux measurements are highly sensitive and allow quantification of even very small fluxes with good signal-to-noise. However, safety and other issues impose significant restrictions on experimental design.

Here we describe a non-radioactive method to measure transmembrane metal ion flux using Inductively Coupled Plasma Mass Spectrometry (ICP-MS). ICP-MS has been established as the most reliable technique for quantifying metals, metalloids, and some non-metals in a wide range of samples with ease, sensitivity of parts-per-trillion, wide working range of concentrations and low interferences. Since its introduction in the 1980s^1^,^2^, ICP-MS has evolved to become the most sensitive and versatile tool for element-specific detection and quantification. Recent technical improvements that remove spectral interference and increase reproducibility have extended its application complex biological samples^3^,^4^,^5^,^6^,^7^,^8^.

The use of ICP-MS to measure metal ion transport has significant advantages for biological applications. It can be used in a wider range of physiological contexts and can detect metal ions for which no feasible radioactive isotopes exist. Importantly, ICP-MS allows the virtually simultaneous measurement of multiple elements and isotopes in the same tissue sample in a short time. This capability allows the simultaneous monitoring of multiple metal-dependent physiological processes such as coupled and secondary ion transport, and facilitates the study of transport proteins for which the transported ion(s) are not yet identified. In addition, it opens the possibility of using internal or external elemental tags to normalize results in small samples, improving the precision.

As proof-of-principle, we measured Na,K-ATPase transport activity in intact, live mouse skeletal muscles and in human red blood cells (RBCs). The Na,K-ATPase is an essential enzyme in the plasma membrane of all animal cells. It maintains the transmembrane concentration gradients for Na and K ions that underlie membrane excitation, osmotic, and electrolyte balance; and provides the driving force for many secondary transporters that transfer other ions, nutrients and essential metal co-factors across the membrane. We measured its transport activity using naturally abundant rubidium, an established congener for K transport by the Na,K-ATPase^9^. To increase precision, we used the Sulfur content of muscle or the iron content of RBCs, measured simultaneously in each sample, as an elemental index of sample mass in place of tissue wet weight. Sulfur is an abundant metal in muscle tissue where it is present in the cysteine-rich contractile proteins, glutathione, and other proteins. Hemoglobin, the major iron-containing metalloprotein of RBCs, comprises about 35% of RBC wet mass.

## Results

### Reference values for endogenous Rb content in skeletal muscles

Determination of Rb concentration in muscle tissue was possible with a high signal-to-noise ratio and detection levels at low as sub-parts per billion, or sub-nanograms per gram of tissue. Reference values for the endogenous Rb concentration in untreated skeletal muscles was 7,112.3 ± 193.3 ng/g (n = 20) in the mouse extensor digitorum longus muscle (EDL) and 7,371.0 ± 796.3 ng/g based on wet mass of the tissues (n = 10) in the tibialis anterior (TA) muscle. These values were not significantly different and therefore reference levels could be determined from different muscles in the same animal when needed. These values for mouse muscles are in the range of reference values for the Rb content of bovine skeletal muscle (NIST RM 8414; see Methods) and were subtracted from measurements of the muscle Rb content taken up during active Na,K-ATPase transport.

### Na,K-ATPase transport measured by ICP-MS in skeletal muscles using equimolar replacement of RbCl for KCl

Next, we measured the amount of Rb taken up by quiescent (non-contracting) EDL muscles during a 10 min incubation in a modified physiological buffer in which KCl was replaced on an equimolar basis with 4.7 mM RbCl (Table 1 and Methods). Rb transport rate by the Na,K-ATPase, computed as the ouabain-sensitive component and normalized to muscle wet weight, was 322.0 nMol Rb/g-min at 32 °C. Ouabain is an established, specific inhibitor of Na,K-ATPase transport. The relative standard deviation (RSD) was 35.9% in control and 19.0% in the presence of the inhibitor, ouabain. This transport rate is close to published values for resting transport by the Na,K-ATPase in mouse EDL obtained from measurements of ^86^Rb flux^10^. The RSD is attributed to animal-to-animal differences and the difficulty of obtaining an accurate wet mass of samples weighing only 5–12 mg.

### Muscle Sulfur content as an index of muscle mass

**Figure 1:**
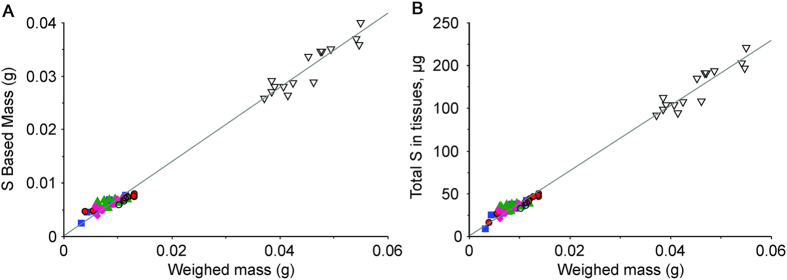
The S content of mouse skeletal muscles is an accurate index of tissue mass. (**a**) total S content of each sample vs. weighed mass. Different symbols represent different muscles and conditions. 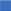, EDL muscles incubated for 10 min in physiological saline containing 4.7 mM RbCl in place of KCl; 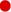, EDL muscles incubated for 10 min in the same solution +1 mM ouabain; 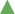 EDL muscles incubated in 200 μM RbCl and 4.7 mM KCl; 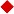 EDL muscles incubated in the same solution +1 mM ouabain; ο, untreated EDL muscle removed from the animal without incubation; 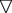, untreated TA muscles. Slope = 3.82 × 10^3^ , correlation coefficient = 0.9868 (**b**) Muscle mass computed from the S content of each sample (S-based mass) vs. weighed mass. Slope = 0.786, correlation coefficient = 0.987.

We tested whether we could reduce the RSD of the measurements by using the muscle Sulfur content as an index of muscle mass. To calculate muscle mass based on Sulfur content, a pooled sample of 20 untreated EDL muscles having a combined wet weight of ~200 mg was used to obtain the average S concentration, average wet weight obtained by weighing the untreated samples, and average dry mass obtained after freeze drying. The average S-to-wet mass ratio of the mouse EDL was 5.49 ± 0.25 mg g^−1^. Using the average S content and an accurate value for the average EDL wet mass, a ratio-to-mass factor, Rb/S, was calculated and used to express the Rb concentration based on S content (S-based mass). The total S content of each sample correlated exactly with the wet mass of the untreated tissues, the same correlation was maintained for EDL and TA, and was independent of sample treatment (Fig. 1a). The S-based mass also correlated linearly with the weighed wet mass of untreated samples (Fig. 1b). However, the slope of 0.80 indicates a 20% decrease when S is used to calculate the sample mass. Muscle is a multicellular tissue and the difference represents the mass contributed by fluid retained in the extracellular spaces during. The S concentrations were identical whether measured using a 10 min or a 1 min incubation (data not shown), indicating that S is not actively transported into or out of the muscle. These results validate the use of S-based mass, obtained from a simultaneous measurement of the S content of each sample, for normalization.

When this normalization is applied to the same samples, the Rb concentrations show a much smaller variation between samples (Table 1, S-based mass). The RSD decreased to 8.0% and 5.2% for control and ouabain-treated samples, respectively. These values are within the expected animal-to-animal variability, indicating that the measurement using S-based mass is not limited by weighing errors. Therefore, the mass determined from S content gives a more precise determination of Rb content with less variability and greater statistical power than obtained using weighed wet mass.

Notably, the net transport rate obtained using S-based mass increased to 399.1 nMol Rb/g-min at 32 °C, indicating that transport activity obtained using wet mass underestimates the actual transport rate of Na,K-ATPase for Rb by ~24%.

### Na,K-ATPase transport measured by ICP-MS in skeletal muscles using tracer RbCl

Given the low detection limits of ICP-MS for Rb, we investigated whether Na,K-ATPase transport could be measured at physiological concentrations of extracellular K, using Rb as a tracer ion (Table 2). Total K uptake determined using a mole fraction of 0.2 mM RbCl to 4.7 mM KCl was 685.0 ± 76.6 (n = 12) ng/g, with a RSD of 11.2%. The slightly increased RSD is due to the much lower Rb concentration in the uptake buffer compared to measurements with equimolar RbCl. Non-specific uptake in the presence of ouabain was 88.4 ± 11.3 (n = 10), with an RSD of 12.8%. However, non-specific uptake of tracer Rb is less than the reference amount in untreated muscles and therefore unreliable. A more reliable estimate of non-specific tracer Rb is the average fractional non-specific uptake obtained using equimolar Rb (26.2% of total uptake; from Table 1).

Net ouabain-sensitive K transport rate by the Na,K-ATPase obtained using tracer Rb and physiological K concentration is 505.5 nMol K/g-min. Notably, this rate is 27% greater (P = 0.01) than that obtained using equimolar Rb as a congener for K, and suggests that the Na,K-ATPase in muscle may transport K more efficiently than Rb.

### Comparison of Na,K-ATPase transport in human RBCs measured by ICP-MS or ^86^Rb

To quantitatively compare Na,K-ATPase transport rates obtained from ICP-MS and ^86^Rb flux in the same preparation, and to test the broader applicability of the method, we measured Rb uptake in human red blood cells by both methods. RBCs are an established model for studies of Na,K-ATPase kinetics and transport cycle^11^. RBCs have a 30–40-fold lower membrane density of Na,K-ATPase than mammalian skeletal muscles and thereby provide a useful model to test the limits of detection of the method. Additionally, use of a homogeneous suspension of single cells allows a direct comparison of wet weight and Fe-based weight without the dilution effect of retained interstitial water.

The endogenous Rb concentration of untreated RBCs was 2,204.7 ± 60.8 ng/g (n = 8 independent units of blood), with a RSD of 2.76% and was subtracted for calculations of Rb uptake by the Na,K-ATPase. The ouabain-sensitive K transport rate of RBCs obtained by ICP-MS under conditions of physiological K concentration and tracer RbCl was 16.0 nMol K/g-min, referred to weighed wet mass (Table 3).

A more precise determination of transport rate was obtained by ICP-MS using the Fe content of RBCs as an index of cell mass. The average Fe content of 8 units of untreated RBCs having an average Hb concentration of 180 g/L was 5,684.14 ± 276.51 ng/g. The total Fe content of each sample correlated exactly with the mass of the untreated RBCs. The average concentration of Rb was not statistically different when calculated by Fe content.

**Figure 2:**
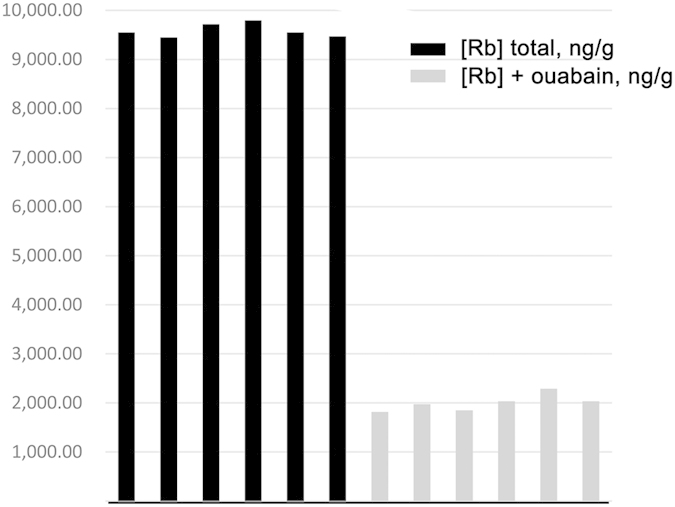
Rb taken up by RBCs in the absence and presence of 1 mM ouabain, measured by ICP-MS and referred to Fe based mass. The RSD for each group was 1.6%.

Na,K-ATPase transport rate measured by ICP-MS using the Fe-based mass of each sample was 18.6 nMol K/g at 37 °C, and the RSD decreased to only 1.6% (Fig. 2 and Table 3). The significantly greater precision obtained using Fe-based mass is attributed to having both the Fe-based mass of the pooled sample before any treatment, and the Fe content of the samples after incubation in uptake buffer. Any loss of cell mass by hemolysis or pipetting during the assay is corrected for since Fe is lost in proportion to cell number; while the Rb content retained in the cells after washing and subtraction of the Fe-based endogenous Rb content reflects Rb taken up by enzyme transport of the remaining intact cells.

The transport rate measured in parallel using tracer ^86^Rb was 11.5 nMol K/g-min based on wet weight. The lower transport rate obtained using tracer ^86^Rb is attributed largely to inaccuracies in obtaining an accurate weight for normalization since, in this case, we have only the initial wet weight of the RBC suspension without any correction for cell loss during the assay. The accuracy of the ^86^Rb tracer measurement could be improved using a larger sample size, a ^86^Rb lot with greater activity, and counting gamma emission directly to avoid sample transfer into scintillant.

## Discussion

Accurate measurement of cellular levels, distribution and flux of metal ions and metal-containing proteins and metabolites is crucial to understanding physiology in both health and disease. Here we describe a method to measure metal ion transport by the Na,K-ATPase in small biological samples using ICP-MS. The method was validated in mouse skeletal muscle, a multicellular tissue, and in suspensions of human red blood cells. The capability of ICP-MS to measure multiple metal ions in the same sample allowed us to use the S content of muscle or the Fe content of RBCs as an elemental index of tissue mass, to increase precision and statistical power.

The K transport rate of Na,K-ATPase measured by ICP-MS in resting mouse EDL muscle was 506 nMol K/g-min at 32 °C, measured with tracer Rb using muscle S content as a mass index. For mouse EDL muscles, which weigh less than 12 mg, conventional normalization to wet weight overestimates muscle mass and thereby underestimates Na,K-ATPase transport rate by about 20%.

A direct comparison of Rb content measured by ICP-MS and tracer ^86^Rb in human RBCs further validated the method in a cell suspension without interstitial spaces. For microliter volumes of RBCs, the greatest precision and accuracy was obtained using ICP-MS with Fe-based mass.

The Na,K-ATPase transport rate of human RBCs under our conditions was 18.6 nMol K/g-min at 37 °C. The low transport rate of human RBCs reflects the low density of Na,K-ATPase in RBC membranes. The specific membrane density of Na,K-ATPase in human RBCs is 1–2 per μm^2 ^12, compared to 200–900 μm^2^ in mammalian skeletal muscle sarcolemma^9^ (assuming a 4–5-fold greater t-tubule membrane area than outer sarcolemma). This transport rate is equivalent to a specific pump turnover rate of 1300–3000/min. The major Na,K-ATPase isoform of human RBCs is the alpha1 isoform. This specific turnover rate is comparable to measurements in other isolated or cultured cell types which predominantly express the same alpha isoform^13^,^14^.

Collectively, these results validate the use of ICP-MS to measure ion transport by the Na,K-ATPase, a vital metal ion membrane transport protein, in small biological samples. Key advantages of ICP-MS are that it allows a wider range of experimental designs and physiological contexts than possible using radioisotopes; and the capability of ICP-MS to measure multiple metal ions in the same sample provides a more accurate index of cell mass for normalization. In future studies, this capability can be exploited further to study the movement of multiple metal ions whose transport is inter-related by coupled exchangers or secondary transporter.

Investigations into the influence of metals on cell and protein function remains a greatly unexplored area of research. The ICP-MS method described here for quantifying metal ion transport by the Na,K-ATPase is easily extended to measurement of other metal ions and transporters, and is widely applicable to measurement of other metal ion-dependent physiological processes. It is applicable to a range of cell types with appropriate adaptation of sample preparation.

## Methods

### Reagents and Standards

All chemicals were trace metal basis grade (Thermo Fisher Scientific, USA; or Sigma-Aldrich, USA). Water of 18 MΩ-cm purity (Milli-Q Academic, EMD Millipore, USA) was used for all solutions. Drinking water certified reference materials were obtained from High-Purity Standards (USA). Elemental standards were obtained from various companies (High-Purity Standards, USA; Claritas PPT, SpexCertiPrep, USA; PlasmaCal, SCP Science, USA; and Thermo Fisher Scientific, USA). The standard reference material RM 8414 (bovine muscle powder) was obtained from the National Institute of Science and Technology (NIST) (USA). Ouabain was from Sigma-Aldrich (USA).

### Animals

C57/B6 mice (The Jackson Laboratory, USA) were used as a source of muscle tissue. Mice were anesthetized (2.5% Avertin, 17 mL/kg) during tissue extraction and euthanized after tissue removal. All procedures were performed in accordance with the Guide For the Care and Use of Laboratory Animals (National Research Council of the National Academies, USA). The experimental protocols were approved by the University of Cincinnati Institutional Animal Care and Use Committee.

### Measurement of basal Rb Uptake in skeletal muscle

The extensor digitorum longus (EDL) muscle was surgically removed and positioned between parallel platinum plate electrodes in a recording chamber perfused with a physiological saline (see buffers, below). One tendon was fixed and the other was attached to an isometric force transducer (BG-50, Kulite Semiconductor Products, Inc., USA) connected to a bridge amplifier (TB-4, WPI, Inc., USA). Two EDL muscles were obtained from each animal. Basal Na,K-ATPase turnover under steady-state conditions was measured as follows. The muscle was: i) perfused in Equilibration Buffer at 32 °C and stimulated with brief pulses to set muscle length to Lo, the length at which the muscle produces maximal twitch force; ii) rested for 15 min in Equilibration Buffer; iii) incubated for 10 min in RbCl -containing Uptake Buffer; iv) immediately washed with cold K-, Rb-, and Na-free Wash Buffer (4 × 15 minute immersions with shaking) to stop enzyme cycling and remove excess cations from the extracellular space; and iv) gently blotted and frozen in acid-washed Eppendorf tubes for subsequent processing by ICP-MS. Non-specific uptake was determined in separate muscles using the identical protocol but with 1 mM ouabain included in all solutions. Untreated muscles were removed, weighed, and processed directly for determination of S content by ICP-MS. Dry mass was determined after freeze drying the muscle.

### Muscle Solutions

The Equilibration Buffer contained (mM): 118 NaCl, 4.7 KCl, 2.5 CaCl_2_, 1.2 MgCl_2_, 1.2 NaH_2_PO_4_, 11 d-glucose, 25 NaHCO_3_; gassed with 95% O_2_, 5% CO_2_; pH 7.4, 32 °C. The Uptake Buffer was identical to Equilibration Buffer except that either 4.7 mM RbCl was used in place of KCl; or, 200 μM RbCl was added as a tracer for KCl. The Wash Buffer contained (mM): 15 Tris-Cl, 2.5 CaCl_2_, 1.2 MgCl_2_, 263 sucrose; pH 7.4, 0–2 °C. Buffers were prepared fresh and used within 1 week. Solutions were perfused through the chamber at 2 ml/min. Temperature was maintained to within ±0.5 °C by an in-line solution heater and controller (Warner Instruments, USA) and monitored by a bath thermistor positioned near the muscle. The content of Rb and S in the equilibration and wash solutions were below the limit of detection by ICP-MS for Rb and 3 ppb for S, while the signal for the samples was in the 2500 and 3500 range. This validates the effectiveness of the wash procedure and allowed us to neglect the trace Rb and S content of the solutions (before addition of exogenous RbCl).

### Measurement of Rb Uptake in human RBCs

Eight units of unfrozen, leucocyte-depleted, packed human RBCs preserved with ADSOL (adenine, dextrose, sorbitol, sodium chloride and mannitol) were obtained from the Hoxworth Blood Center, University of Cincinnati. The units were collected on two dates from healthy volunteers of mixed gender and stored at 4 °C for up to 42 days. The units contained an average 7 × 10^9^ RBC/ml and 180 g Hb/L. All procedures were approved by the Institutional Biosafety Committee of the University of Cincinnati. Prior to experiments, an aliquot was taken, washed to remove plasma, and re-suspended in a Rb-free RBC Buffer containing (mM): 130 NaCl, 5 KCl, 2 CaCl_2_, 1 MgCl_2_, 0.5 NaH_2_PO_4_, 10 d-glucose, 12 NaHCO_3_, 10 HEPES; pH 7.4 at 37 C, 290 mOs/kg H_2_O. The wash consisted of 3 cycles of dilution in 5 volumes of buffer followed by centrifugation at 500 × g for 10 min at 4 C, and removal of supernatant. After the final wash, the sample was re-suspended in buffer at a hematocrit of 0.5.

Uptake Buffer was prepared by adding 240 μM RbCl to the RBC Buffer. Rb uptake was measured under 4 conditions in tubes containing: 150 μl of washed and suspended RBCs, 50 μL of either 10 mM ouabain (1 mM final concentration) or H_2_O, 800 μL of Uptake Buffer, and 0 or 4 nCi/ml ^86^Rb. Uptake was carried out for 2 h at 37 ± 1 °C. Each condition was measured in triplicate and all samples were processed in parallel.

After the incubation, samples were washed 3 times as described above to remove extracellular Rb and/or ^86^Rb. After the final wash, the sample was re-suspended in H_2_O and taken either for counting, or determination of Rb by ICP-MS. ^86^Rb activity was determined by liquid scintillation counting of beta decay (Filter-Count, Perkin-Elmer, USA; LS-6500 Liquid Scintillation Counter, Beckman, USA). Aliquots of Uptake buffers without and with ^86^Rb were taken in each experiment and used to obtain a calibration factor in cpm/nMole of Rb. ICP-MS determination of Rb and Fe concentrations was carried out as described for skeletal muscle samples.

The content of Rb in the initial, nominally Rb-free, RBC buffer and in the final supernatant was below the limit of detection by ICP-MS for Rb, while the signals for the samples were in the 12,000 ppb range. This validates the effectiveness of the wash procedure and allowed us to neglect the trace Rb content of the RBC buffer.

### Sample preparation for ICP-MS-MS Total Metal Analysis

Muscle samples were subjected to acidic mineralization to oxidase the organic matter, solubilize all metals and simplify the matrix. The samples were weighed on an analytical balance and placed in acid-washed 10 ml glass digestion vials with Teflon-lined caps, to which 1 ml of 1:1 HNO_3_:H_2_O and 0.1 ml of internal standard mix were added. A pre-digestion step was carried out for 1 h at 120 °C using a heating block. Sample volumes were brought to 2 ml with doubly deionized water and a microwave assisted digestion was performed (CEM Corp., USA). The microwave program consisted of a ramp to 120 °C in 5 min and a holding time of 30 seconds; followed by a ramp to 190 °C in 5 min and holding time of 7 minutes. Once the samples were mineralized, sample volumes were brought to 10 ml with doubly deionized water. A standard reference material, bovine muscle powder (NIST RM 8414, 0.0070 g–0.0100 g) was treated identically and assayed to establish the accuracy of the measurement. The concentration of all metals in the reference material were within the NIST range.

For RBC analysis, the final volume after the Rb uptake experiment was digested. For this the samples were analytically transferred to a glass digestion vial by using two portions of 1 ml of doubly deionized water to rinse the original vial. All transferred volume was digested after adding 1 ml of concentrated trace metal grade nitric acid and 0.4 ml of internal standard mix. The mixture was heated to 140 °C for 5 h in a heating dry bath. 250 μl of H_2_O_2_ were added to complete the digestion and heated for 1 h at 140 °C. After complete mineralization the samples were brought to 40 ml by adding doubly deionized water before the analysis by ICP-MS.

### Quantification of elemental content by ICP-MS

The selected elements -Na, Mg, P, S, K, Ca, Mn, Fe, Ni, Cu, Zn, Rb, & Pb were determined by ICP-MS by the external calibration method, using an Agilent 8800 inductively coupled plasma mass spectrometer triple quadrupole (ICP-MS QQQ, Agilent Technologies, USA) equipped with a micro concentric nebulizer, (Meinhard MicroMist, Meinhard, USA), a peltier cooled double pass spray chamber, standard torch, and auto sampler. Data analysis was performed using Mass Hunter workstation version 4.1 for ICP-MS QQQ software (Agilent Technologies, USA) to determine the total concentration of elements in the tissues and solutions. Two tune modes were used sequentially to ensure proper ionization and interference removal (Table 4). Internal standards for Sc, Y, In, & Ce were used to represent the full mass range. Results are given as the mean and RSD of measurements from independent samples.

Instrument calibration curves for detection of Rb in an NIST standard sample of bovine skeletal muscle were linear with regression coefficient of 1.0000 in the range of 0.1–400 ppb. In no gas mode, the instrument limit of detection is 1 femtogram ml^−1^, or 6 ppt. This implies a detection limit of 6 femtograms or 0.07 femtomoles of Rb per milligram wet tissue. The calibration was linear for all detection modes.

### Detection of S signals without isobaric interference

The ICP-MS signal for Rb is not compromised by isobaric interferences, but this is not the case for S. Nitrogen, oxygen and hydrogen-based polyatomic interferences from atmospheric plasma represent a major challenge for direct analysis of S at its most abundant isotope. High resolution instruments can be used to solve this problem, but they are costly and not as robust as the quadrupole based low resolution instruments. Helium is commonly used to remove the majority of the ^34^S interferences, but an important loss of sensitivity is observed^1^. A reaction with oxygen before the quadrupole has also been employed, as the polyatomic interferences do not react with oxygen, but the reaction is matrix-dependent and the analytes at the oxidized S product become interferents. A new approach was developed in early 2013 by Agilent Technologies which consists of the addition of an extra quadrupole that allows filtering out all ions except the m/z = 32 (S, and interferences), and then reacting them with oxygen to filter out the original m/z, which is now conformed by the un-reacted polyatomic interferents. This new technology allows the quantification of S with less interference without signal loses, resulting in lower limits of detection and background signal. Although we used this technology for the Rb transport assay described here, we note the very intense signals of S in skeletal muscles of the studied size would also allow the use of helium in collision mode to remove S interferents.

### Statistical Analysis

Origin 8.5 was used to perform all statistical analysis. The comparisons between muscle samples, un-treated EDL vs. TA, were performed with a t- test. The comparison of Rb values between Rb treated and Rb + Ouabain treated samples were made with a t-test, and showed a p < 0.01 with a power of 0.97 for the reported sample sizes.

## Additional Information

**How to cite this article**: Figueroa, J. A. L. *et al.* Metal ion transport quantified by ICP-MS in intact cells. *Sci. Rep.* **6**, 20551; doi: 10.1038/srep20551 (2016).

## Figures and Tables

**Table 1:** Rb uptake by the Na,K-ATPase in quiescent mouse EDL muscle measured by ICP-MS using equimolar replacement of RbCl for KCl in the uptake buffer.

	Normalized to wet weight			Normalized to S-based mass		
	Rb content *ng/g*	*RSD %*	Rb transport rate *nMol Rb/g-min*	Rb content *ng/g*	*RSD %*	Rb transport rate *nMol Rb/g-min*
no ouabain (n = 10)	397,135.9 ± 142,548.5	*35.9*	467.2	459,860.8 ± 36,809.4	*8.0*	541.0
+ouabain (n = 5)	123,431.5 ± 23,393.8	*19.0*	145.2	120,601.2 ± 6,239.8	*5.2*	141.9
*NET transport by Na,K-ATPase*			***322.0***			***399.1***

Rb transport rate was measured using a 10 min incubation in physiological saline containing 4.7 mM RbCl in place of KCl, and nominally 0 mM KCl, at 32 °C. Non-specific Rb uptake contributed by all other K and Rb transport pathways was measured in independent muscles using 1 mM ouabain. Using S-based muscle mass, non-specific Rb uptake was 26.2% of total uptake, and was subtracted to obtain net ouabain-sensitive uptake by the Na,K-ATPase. *RSD*, relative standard deviation. Rb content was normalized either to weighed wet tissue mass or to the S-based mass.

**Table 2:** Rb uptake by the Na,K-ATPase in quiescent mouse EDL muscle measured by ICP-MS using 200 μM RbCl as tracer.

	Normalized to wet weight				Normalized to S-based mass			
	Rb content *ng/g*	*RSD %*	Rb transport rate *nMol Rb/g-min*	K transport rate *nMol K/g-min*	Rb content *ng/P*	*RSD %*	Rb transport rate *nMol Rb/g-min*	K transport rate *nMol K/g-min*
no ouabain (n = 12)	18,959.8 ± 6,928.5	*36.5*	22.3	524.2	24,776.4 ± 2,769.3	*11.2*	29.2	685.0
+ouabain (n = 10)	2,699.2 ± 1,355.5	*50.2*	3.18	74.6	3,198.3 ± 409.8	*12.8*	3.8	88.42
*NET transport by Na,K-ATPase*				***449.6***				***505.5***

Rb uptake was measured using a 10 min incubation in Uptake Buffer containing 4.7 mM KCl and 200 μM RbCl, at 32 C. Ouabain-sensitive Na,K-ATPase transport was obtained as described in Table 1. The amount of Rb taken up by the muscle was scaled by the molar ratio of K to Rb in the Uptake Buffer (23.5) to obtain the transport rate of the Na,K-ATPase for K. NET ouabain-specific transport by Na,K-ATPase was obtained after subtracting the fractional non-specific uptake computed as 26.2% of total uptake (from Fig 1) as described in text.

**Table 3:** Comparison of Rb transport by human RBCs measured by ICP-MS and ^86^Rb tracer.

	ICP-MS						^86^Rb tracer		
	by wet weight			Fe- based mass			by wet weight		
	Rb content *nMol Rb*/*g*	*RSD %*	K transport rate *nMol K*/*g-min*	Rb content *nMol Rb*/*g*	*RSD %*	K transport rate *nMol K*/*g-min*	Rb content *nMol Rb*/*g*	*RSD %*	K transport rate *nMol K*/*g-min*
no ouabain (n = 6)	79.0 ± 6.8	*8.6*	21.2 ± 1.8	112.8 ± 1.6	*1.6*	23.5 ± 0.4	78.5 ± 15.0	*19.1*	16.4 ± 3.1
+ouabain (n = 6)	15.1 ± 1.2	*8.2*	5.22 ± 0.3	23.5 ± 2.0	*1.6*	4.9 ± 0.1	23.4 ± 2.3	*9.9*	4.9 ± 0.5
NET transport by Na,K-ATPase		**16.0**			**18.6**			**11.5**	

All measurements were run in parallel under identical conditions using the same pooled batch of RBCs. The batch was pooled from 4 units of leucocyte-depleted RBCs, washed, and re-suspended in Rb-free buffer at a hematocrit of 50%. The incubation mix contained 150 μL RBCs, 5 mM K, 200 μM RbCl, and Uptake Buffer without or with 1 mM ouabain, in a volume of 1 ml. Radioisotope tracer assays contained, in addition, 4 nCi/ml ^86^Rb. Uptake was carried out for 2 h at 37 C. K transport rate was computed by multiplying the Rb transport rate by the molar ratio of K to Rb in the Uptake buffer. The endogenous Rb concentration of the pooled, untreated RBCs was 2,374 ng/g and was subtracted in all calculations of exogenous Rb uptake.

**Table 4:** Instrument tune parameters for ICP-MS QQQ.

No gas mode		He mode		O_2_ mode	
Forward power	1450 W	Forward power	1500 W	Forward power	1600 W
Nebulizer gas flow	1.0 L/min	Nebulizer gas flow	1.0 L/min	Nebulizer gas flow	1.0 L/min
Extract 1	0.0 V	Extract 1	0.5 V	Extract 1	0.0 V
Extract 2	−140 V	Extract 2	−165.5 V	Extract 2	−200.0 V
Isotopes monitored	^23^Na, ^24^Mg, ^39^K, ^43^Ca, ^44^Ca, ^45^Sc, ^85^Rb & ^87^Rb	Isotopes monitored	^23^Na, ^24^Mg, ^45^Sc ^43^Ca, ^44^Ca, ^55^Mn, ^56^Fe, ^60^Ni, ^63^Cu, ^66^Zn, ^85^Rb, ^87^Rb & ^208^Pb	Isotopes monitored	^31^P → ^31^P^16^O, ^32^S → ^31^S^16^O
		Helium gas flow	3 ml min^−1^	Oxygen gas flow (0.3 ml min^−1^)	30%
OctP Bias	−8.0 V	OctP Bias	−18.0 V	OctP Bias	−5.0 V
OctP RF	150 V	OctP RF	150 V	OctP RF	200 V
Energy discrimination	5.0 V	Energy discrimination	5.0 V	Energy discrimination	−7.0 V
